# Scientific Items

**Published:** 1887-02

**Authors:** 


### SCIENTIFIC ITEMS.

**The Resistance of the Atmosphere.**—From Professor Langley's illustrated paper on "Comets and Meteors," in the January *Century*, we quote as follows: "Everybody has noticed that if we move a fan gently, the air parts before it with little effort, while, when we try to fan violently, the same air is felt to react; yet if we go on to say that if the motion is still more violent, the atmosphere will resist like a solid, against which the fan, if made of iron, would break in pieces, this may seem to some an unexpected property of the 'nimble' air through which we move daily. Yet this is the case, and if the motion is only so quick that the air can not get out of the way, a body hurled against it will rise in temperature like a shot striking an armor plate. It is all a question of speed, and that of the meterorite is known to be immense. One has been seen to fly over this country from the Mississippi to the Atlantic in an inappreciably short time, probably in less than two minutes; and though at a presumable height of over fifty miles, the velocity with which it shot by gave every one the impression that it went just above his head, and some witnesses of the unexpected apparition looked the next day to see if it had struck their chimneys. The heat developed by arrested motion in the case of a mass of iron moving twenty miles a second can be calculated, and is found to be much more than enough, not only to melt it, but to turn it into vapor; though what probably does happen is, according to Professor Newton, that the melted surface-portions are wiped away by the pressure of the air and volatized to form the luminous train, the interior remaining cold, until the difference of temperature causes a fracture, when the stone breaks and pieces fall—some of them at red-hot heat, some of them possibly, at the temperature of outer space, or far below that of freezing mercury.

"Where do these stones come from? What made them? The answer is not yet complete; but if a part of the riddle is already yielding to patience, it is worthy of note, as an instance of the connection of the sciences, that the first help to the solution of this astronomical enigma came from the chemists and the geologists." —*National Druggist.*

**The Purity of Mid-Atlantic Air.**—In the course of an address on the Action of Micro-Organisms on Surgical Wounds, Professor F. S. Dennis, of New York, states that during his last trip across the Atlantic he made some experiments to test the purity of the air about 1000 miles from land. He employed capsules of sterialized gelatine, and exposed them for fifteen minutes. One capsule was exposed in the state-room upon the main deck of the steamer. Within eighteen hours over 500 points of infection had developed. Two capsules exposed in a similar manner in a cabin on the promenade deck, where the circulation of air was free, showed five or six points of infection each ten days afterwards. A capsule exposed over the bow of the ship was found to be entirely uncontaminated. These experiments are on the same lines as those of Pasteur and Tyndall upon the mountain air of Switzerland, and, so far as they go, they show the germless condition of mid oceanic air, and also the need for much more efficient ventilation in the state-rooms of even the first-class American liners.—*Medical and Surgical Reporter.*

**Electric Lamp.**—Dr. Fleming says an incandescent lamp is not only a useful thing, but it has about it many points of great interest in physics. Many persons had the impression that the interior of a glow lamp was a place that was empty of all air particles, but this was not the case; it was as full as it well could be. Maxwell had shown that in a small cube of 1-100,000 of an inch there would be found 100,000,000 molecules of ordinary air, so that in a cubic inch of air there were a number of molecules represented by 100,000,000,000,000,000. In a Swan lamp when exhausted to one-millionth of an atmosphere, there remained some 400,000,000,000 molecules of air. As it took about ten days to count a million, a simple calculation would show that to count the number of molecules in such a vacuum would take 120,000 years of continual counting.—*Scientific American.*

**Impure Ice.**—The New York State Board of Health, in a report on the dangers of contaminated ice, draws the following conclusions: Ice formed in impure water has caused sickness; it may contain from 8 to 10 per cent. of the organic matter dissolved in the water, and in addition a very large amount of the organic matter that had been merely suspended or floating in it; it may contain living animals and plants ranging in size from visible worms down to the minutest spores, and the vitality of these organisms may be unaffected by freezing.—*Journal Med. Association.*

**According** to the report of the National Telephone Association telephony has not yet reached such a point as to make long-distance service formidable in competition with telegraphy. One hundred miles or so seem to be the paying limit at present, notwithstanding that there are several lines which exceed that distance. That of the Wisconsin Telephone Company is the longest, and has 199 miles in regular and successful operation.—*Chicago Inter-Ocean.*

**Scientific Fish Story.**—An Italian has discovered that fishes are fond of music. To one Signor Garetti the honor of the discovery is said to be due; and recently, with a party of friends, he is said to have tried the experiment on Lake Geneva, which proved quite successful. Musical notes, especially those produced by the human voice, attracted the fishes in great numbers around the boat. Fishermen should try the experiment.—*Idid.*

